# Expression of concern: Effects of LncRNA-HOST2 on cell proliferation, migration, invasion and apoptosis of human hepatocellular carcinoma cell line SMMC-7721

**DOI:** 10.1042/BSR-20160532_EOC

**Published:** 2020-08-25

**Keywords:** Hepatocellular carcinoma, Human ovarian cancer specific transcript 2, Long noncoding RNA, Migration, Proliferation, SMMC-7721 cell line

The Editorial Office has been made aware of potential issues surrounding the scientific validity of this paper, hence has issued an expression of concern to notify readers whilst the Editorial Office investigates.

